# Distribution of Functional Liver Volume in Hepatocellular Carcinoma Patients with Portal Vein Tumor Thrombus in the 1st Branch and Main Trunk Using Single Photon Emission Computed Tomography—Application to Radiation Therapy

**DOI:** 10.3390/cancers3044114

**Published:** 2011-10-31

**Authors:** Shintaro Shirai, Morio Sato, Yasutaka Noda, Kazushi Kishi, Nobuyuki Kawai, Hiroki Minamiguchi, Motoki Nakai, Hiroki Sanda, Shinya Sahara, Akira Ikoma, Tetsuo Sonomura

**Affiliations:** Department of Radiology, Wakayama Medical University, 811-1 Kimiidera, Wakayamashi, Wakayama 641-8510, Japan; E-Mails: shshirai@wakayama-med.ac.jp (S.S.); y-kiyose@wakayama-med.ac.jp (Y.N.); Kazushi.kishi@gmail.com (K.K.); nobkawai@wakayama-med.ac.jp (N.K.); hiromina4@hotmail.com (H.M.); momonga@wakayama-med.ac.jp (M.N.); sandabrid88@ybb.ne.jp (H.S.); s-sahara@wakayama-med.ac.jp (S.S.); yfb04322@nifty.com (A.I.); sonomura@wakayama-med.ac.jp (T.S.)

**Keywords:** hepatocellular carcinoma, portal vein tumor thrombus, radiotherapy, radiation-induced liver disease, single photon emission computed tomography

## Abstract

**Purpose:**

To analyze the distribution of functional liver volume (FLV) in the margin volume (MV) surrounding hepatocellular carcinoma (HCC) with portal vein tumor thrombus (PVTT) before radiation therapy (RT) and to verify the safety of single photon emission computed tomography-based three-dimensional conformal radiotherapy (SPECT-B3DCRT) by exploring the relation of FLV in MV to radiation-induced liver disease (RILD).

**Methods and Materials:**

Clinical target volume (CTV) included main tumor and PVTT, and planning target volume (PTV) included CTV with a 10 mm margin. MV was defined as PTV–CTV. FLV ratio in MV was calculated as FLV in MV/MV × 100 (%). The two high-dose beams were planned to irradiate FLV as little as possible. Fifty-seven cases of HCC (26/57, 46%; Child–Pugh grade B) with PVTT underwent SPECT-B3DCRT which targeted the CTV to a total dose of 45 Gy/18 fractions. The destructive ratio was defined as radiation induced dysfunctional volume/FLV × 100 (%).

**Results:**

We observed a significant negative correlation between FLV ratio in MV and CTV (p < 0.001). Three cases with CTVs of 287, 587 and 1184 cm^3^ experienced transient RILD. The FLV ratio in MV was highest in patients with RILD: nine patients with CTV of 200–300 cm^3^, three with CTV of 500–600 cm^3^, and two with CTV of 1100–1200 cm^3^. The destructive ratio yielded a mean value of 24.2 ± 1.5%.

**Conclusions:**

Radiation planning that takes into account the distribution of FLV appears to result in the least possible RILD.

## 1. Introduction

More than a quarter of a century has passed since Yamada *et al.* applied transcatheter arterial chemoembolization (TACE) for unresectable hepatocellular carcinoma (HCC) with portal tumor thrombus (PVTT), a technique that caused severe liver damage and had a poor prognosis [1]. The difficult problem of improving the prognosis of patients with unresectable HCC with PVTT is as yet unresolved [2], mainly because the pathophysiology of liver containing HCC with PVTT is poorly understood. We would expect interruption of portal venous flow by PVTT to affect liver function; nonetheless, there is no documentation of analysis of liver function in the presence of PVTT, except our previous reports using single photon emission computed tomography (SPECT) with ^99m^Tc galactosyl human serum albumin (^99m^Tc-GSA) [3,4].

Our analyses were conducted based on the findings of two previous studies: that of Shuke *et al.* [5], who found that ^99m^Tc-GSA was superior to other radioisotopes for analyzing local liver function; and that of Nanashima *et al.* [6], who found that SPECT with ^99m^Tc-GSA was superior to enhanced computed tomography (CT) using contrast medium for evaluating liver function. We classified HCC patients with PVTT into localized type (mainly corresponding to main tumor and PVTT surrounded by functional liver) and wedge type (corresponding to main tumor, PVTT, and dysfunctional liver) according to SPECT-delineated areas of non-accumulation [3]. Qualitative analysis after SPECT-based three-dimensional conformal radiotherapy (SPECT-B3DCRT) revealed no significant difference between mean dose to normal liver and mean dose to functional liver for the localized type, but a significant difference between these two doses for the wedge type [3]. SPECT-B3DCRT brought about that the median survival time of the wedge type HCC with PVTT and the HCC sized 8 cm or greater with PVTT was 13.5 months and 10.3 months, respectively, which was comparable to 13.6 months in the surgical hepatectomy series [3,4]. The follow-up SPECT images after radiation therapy (RT) revealed that radiation-induced dysfunctional liver volume (RIDFLV) was mainly observed in the margin volume (MV) surrounding the main tumor and PVTT [4].

Therefore, we conducted the present study with the aim of analyzing the distribution of functional liver volume (FLV) in the MV surrounding HCC with PVTT before RT, and of verifying the safety of SPECT-B3DCRT by exploring the relation of FLV in MV to radiation-induced liver disease (RILD) [7].

## 2. Methods and Materials

### 2.1. Patients and Tumor Characteristics

This prospective clinical investigation was approved by the Ethics Committee of our institute, and informed consent was obtained from all patients. The study procedures were in accordance with the Helsinki Declaration of 1975, as revised in 2000. The eligibility criteria comprised the following: (1) unresectable HCC with PVTT in the 1st branch and/or the main trunk; (2) no limit to HCC size; (3) the absence of extrahepatic metastasis; (4) no ascites or under medical control of ascites; (5) Child–Pugh grade A or B; and (6) performance status of between 0 and 2 on the Eastern Cooperative Oncology Group scale. As previously reported [3,4], TACE was performed in each patient using 3–10 mL lipiodol (Guerbet, Charles de Gaulle, France) with or without gelatin sponge particles (Spongel, Yamanouchi, Tokyo, Japan), within two months after radiation treatment and at follow-up if intrahepatic metastases recurred.

### 2.2. Identification of FLV

FLV was identified prior to radiation planning. For accuracy, Tc-99m-GSA SPECT, dynamic contrast-enhanced CT, and simulation CT were performed within no longer than two weeks before 3DCRT. To enable accurate fusion of the images, all images of the whole liver in each modality were obtained with 5-mm thickness under breath holding at end-expiration. We contoured the whole liver, main tumor, PVTT, and other hepatic tumors (intrahepatic metastases) on the dynamic CT images of each patient. We defined normal liver volume (NLV) as the area remaining after subtracting main tumor plus PVTT plus other hepatic tumors from the whole liver. Within NLV, areas that had the same radioisotope (RI) filling defects as that of HCC were defined as dysfunctional liver volume (DFLV); areas of hyper-accumulation relative to HCC were defined as FLV [8]. The corresponding areas on SPECT images were then outlined on the dynamic CT images, and these transferred areas were fused with the corresponding simulator CT images and outlined.

### 2.3. Radiation Planning and Radiation Therapy

The previously described 3DCRT method [3,4] is briefly summarized as follows. All patients underwent 3DCRT in the supine position with both arms raised above the head. We used no special apparatus for respiratory immobilization. To minimize the effects of respiration, the subjects practiced breath holding for 10–15 seconds at the time of end-expiration until the position could be maintained within 5 mm under X-ray fluoroscopic monitoring. RT using a 10 MV linear accelerator was repeatedly delivered during breath holding at end-expiration for 10–15 seconds at a time.

**Figure 1.**
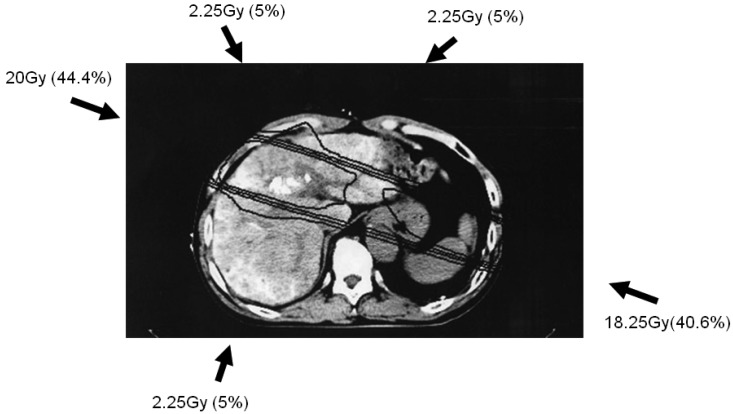
Simulation planning of three-dimensional conformal radiotherapy. Five radiation beams (arrows) were set up to irradiate the clinical target volume (CTV), defined as main tumor and portal vein tumor thrombus. Doses of the high-dose beams were limited to 38.25 Gy/18 fractions/4 weeks; three additional low-dose beams of 6.75 Gy/18 fractions/4 weeks were required to elevate the dose to the CTV, resulting in a total dose of 45 Gy. The iso-dose distribution curves indicate 40, 30, 20, and 10 Gy, from innermost to outermost.

Simulation CT data were transferred to a three-dimensional radiation treatment planning system (Pinnacle, ADAC Laboratories, Milpitas, CA, USA); subsequent CT treatment planning is shown in Figure 1. Clinical target volume (CTV) was defined as main tumor plus PVTT. Planning target volume (PTV) included CTV with a 10-mm margin, accounting for respiratory-induced motion, penumbra covering, and variations in daily setup.

**Figure 2.**
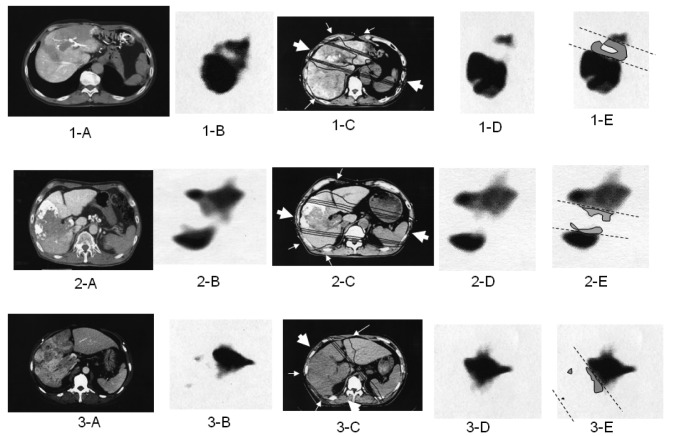
Computed tomography (CT; 1-A, 2-A, and 3-A) prior to three-dimensional conformal radiation treatment (3DCRT) in three cases of hepatocellular carcinoma associated with portal vein tumor thrombi. Subfigures 1-B, 2-B, and 3-B show single photon emission computed tomography (SPECT). Case 1 is classified as localized type because the filling defect is localized by a surrounding area of high accumulation; Case 2 is classified as wedge type because the area of radioisotope (RI) filling defect is in a wedge form that separates areas of high accumulation; and Case 3 is classified as extensive wedge type because the area of RI filling defect occupies almost a whole lobe. Subfigures 1-C, 2-C, and 3-C show four iso-dose lines from simulation CT during radiation planning (from innermost to outermost: 40, 30, 20, and 10 Gy). The large arrows indicate high-dose beams of 38.25 Gy; small arrows indicate low-dose beams of 6.75 Gy. Subfigures 1-D, 2-D, and 3-D show SPECT at 2 months after 3DCRT. New filling defect lesions are evident compared with Subfigures 1-B, 2-B, and 3-B; we termed these lesions radiation-induced dysfunctional liver (RIDFL). They are shown as gray areas on Subfigures 1-E, 2-E, and 3-E, and are located within the 20 Gy iso-dose curve.

The optimal 3DCRT beam directions (optimal angles of the gantry) were explored using the SPECT images (Figure 2; 1-B, 2-B, 3-B) for guidance. The directions of the two high-dose beams were designed to cover primarily the main tumor and PVTT, and to irradiate FLV as little as possible. As shown in Figure 1, the doses of the high-dose beams were limited to 38.25 Gy/18 fractions/4 weeks to prevent adverse effects to the duodenum, spinal cord, and kidneys [9,10]. Three additional low-dose beams of 6.75 Gy/18 fractions/4 weeks were required to elevate the dose to the CTV, resulting in a total dose of 45 Gy to the isocenter [11]. These low-dose beams were designed to avoid irradiating risk organs such as the stomach, duodenum, and spinal cord. Doses of 2.25 Gy for each additional beam do not cause liver damage even if the beams irradiate FLV. In terms of the kidney, the volume irradiated at 20 Gy or more was planned to not exceed 30% of the total volume. Finally, the couch angle was adjusted to the maximum limit of 90°.

### 2.4. FLV in the Margin Area before RT

Because RIDFLV was observed after SPECT-B3DCRT in the area surrounding CTV, we performed analysis of liver function in the area surrounding CTV before RT using a quantitative indicator. We defined margin volume (MV) as the area located 1 cm outside the CTV, or PTV–CTV. To evaluate the relation between FLV in MV and the size of main tumor plus PVTT (CTV), we calculated the FLV ratio in MV as follows: FLV ratio in MV = FLV in MV/MV × 100 (%).

### 2.5. Follow-up Evaluation

All patients underwent follow-up CT one month after RT. Follow-up SPECT using Tc-99m-GSA was performed two months after completion of SPECT-B3DCRT, and was used to determine RIDFLV (Figure 2; 1-E, 2-E, 3-E). Laboratory tests for blood and liver function were conducted weekly during SPECT-B3DCRT, monthly for four months after the completion of SPECT-B3DCRT, and several times after TACE. If changes in transaminase or other factors levels were detected, the tests were rechecked after an interval of 1–2 weeks.

### 2.6. Radiation-Induced Liver Disease (RILD)

It is important to differentiate RILD from adverse effects of TACE within two months after 3DCRT. In analyzing RILD to exclude the negative effects of TACE, we used the same method as that proposed in our previous manuscript [4]: transaminase elevation, which is one adverse effect of TACE, is clearly characterized by occurring immediately after TACE and having a short duration (median, five days) [1]. Fever and abdominal symptoms are generally associated with this period, with high incidence (76%–88%) [1]. RILD lacks marked symptoms in the early stages [4]. Based on differences in the clinical symptoms, occurrence, and duration of transaminase elevation, RILD is easily differentiated from the adverse effects of TACE.

All patients were evaluated for evidence of RILD within four months after RT completion. We assessed any evidence of RILD according to the endpoints of RILD described by Pan *et al.* [7]. Namely, classic RILD involves anicteric hepatomegaly and ascites. Classic RILD also involves elevated alkaline phosphatase (more than twice the upper limit of normal or baseline value). Nonclassic RILD involves elevated liver transaminase (more than five times the upper limit of normal) or CTCAE Grade 4 levels in patients with baseline values more than five times the upper limit of normal within three months after completion of RT, or a decline in liver function (measured by a worsening of Child-Pugh score by 2 or more), in the absence of classic RILD (Table 1).

### 2.7. Analysis of FLV ratio in MV and RILD

A large amount of damage to liver parenchyma would lead to RILD even if HCC sized less than 5 cm were exposed to irradiation [12]. As the combined size of HCC plus PVTT increases [4], the CTV itself becomes a matter of concern. It is expected that FLV in MV is destroyed by 3DCRT. We compared the FLV ratio in MV with CTV in patients with HCC plus PVTT in a correlation scatter distribution diagram, and identified cases of RILD from the diagram. We also compared the backgrounds and 3DCRT characteristics of patients with and without RILD. Furthermore, RIDFLV and RIDFLV/FLV (%) were compared between these two groups.

### 2.8. Estimation of RIDFLV and Destruction Ratio

**Figure 3.**
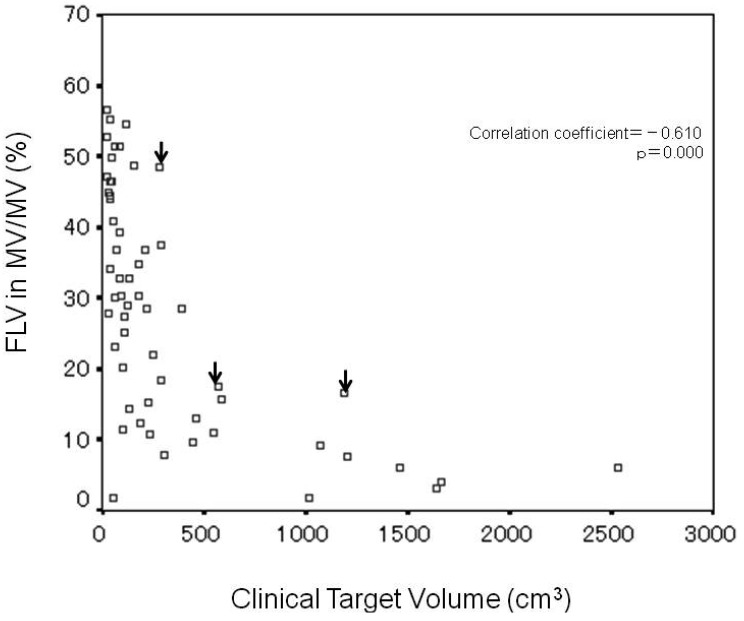
Scatter diagram of all patients showing the correlation between clinical target volume (CTV) and functional liver volume ratio in margin volume. A significant negative correlation is seen (correlation coefficient = −0.610, p < 0.001). The three cases of radiation-induced liver disease (arrows) are at the upper margin of patients with similar CTVs.

We previously reported the occurrence of RIDFLV after RT [3,4]. The presence of RIDFLV was confirmed in all patients who received follow-up SPECT. In the present study, we performed quantitative analysis of RIDFLV. Namely, as illustrated in Figure 2, the follow-up SPECT images (Figure 2, 1-D, 2-D, 3-D) were fused with the corresponding simulation CT images (Figure 2, 1-C, 2-C, 3-C) and the image data were input to the RT planning computer. Because the simulation CT images included the SPECT image prior to RT, RIDFL was outlined by subtracting the SPECT image obtained after RT from that prior to RT (Figure 2, 1-E, 2-E, 3-E) and RIDFLV was calculated using the data of all slices; this value was then used to calculate the destruction ratio as follows: Destruction Ratio = RIDFLV/FLV × 100 (%). RIDFLV and the destruction ratio are outcome factors rather than predictive factors, and thus cannot be used prior to RT to predict liver damage.

We then explored predictive factors of liver damage caused by RT, based on the finding that the borderline (dashed lines in subfigures E in Figure 2) between RIDFL and the remaining FL after 3DCRT is almost linear, and that the four iso-dose curves on the simulation CT are also linear, and almost correspond with the outer line of the high-dose beam. Therefore, the predictive factors of liver damage caused by RT for (X) Gy could be determined by calculating the relationship of the iso-dose curve to the dashed line, as FLV_X_Gy = RIDFLV/FLV × 100 (%). We fused the simulation CT and the follow-up SPECT to determine which Gy isodose curve was coincident with the dashed line.

### 2.9. Analysis of Risk Factors Related to RILD

We compared backgrounds and 3DCRT characteristics between patients with and without RILD. The subject factors for univariate analysis were gender, age, performance status, apex of PVTT, hepatitis viral infection, AFP, Child–Pugh class, CTV, NLV, FLV, RIDFLV, and RIDFLV/FLV (%).

### 2.10. Statistical Methods

Student's *t*-test was used to compare continuous variables between patients with and without RILD. Fisher's exact test was used to compare categorical variables between patients with and without RILD. Correlation between CTV and FLV in MV/MV (%) was analyzed using Pearson's correlation analysis and Pearson's correlation coefficient. For each analysis, p < 0.05 was considered statistically significant.

## 3. Results

Between January 2005 and December 2008, 61 patients underwent SPECT-based-3DCRT for HCC with PVTT of the 1st branch and/or main portal trunk. The data were analyzed in October 2010. Of the 61 cases, four patients with progressive disease (PD) were withdrawn from the study: follow-up CT at one month after RT demonstrated rapid progress of intrahepatic metastases and these patients did not receive follow-up SPECT or scheduled TACE because of poor condition. Two of these patients died of esophageal varices rupture at 56 and 68 days after RT, and the other two died of tumor growth at 44 and 98 days after commencement of RT.

The remaining 57 patients were enrolled in the study. Median follow-up was 9.0 months (range, 3.8–66.5 months). In all patients, RT was completed and adequate SPECT images were acquired before and two months after RT. In three patients with Child-Pugh B before RT, RILD was observed 27, 36, and 39 days after RT as a 2-point worsening of Child–Pugh score. TACE was not conducted before the onset of RILD. These patients received intravenous drip infusion of aminolevan to protect liver function, and RILD improved at 23, 8, and 34 days after onset, respectively. No symptoms related to hepatic toxicity were observed, and these patients subsequently received the scheduled TACE. Patient backgrounds and tumor characteristics are listed in Table 2.

### 3.1. Correlation between CTV and FLV Ratio in MV

Analysis of the correlation between CTV and FLV ratio in MV (%) revealed significant negative correlation (correlation coefficient = −0.610, p = 0.000) (Figure 3), and that the FLV ratio in MV decreased with increasing CTV. In the three cases of RILD, indicated by arrows on the figure, CTV and the FLV ratio in MV were 287 cm^3^ and 48.5%, 587 cm^3^ and 17.6%, and 1184 cm^3^ and 16.6%, respectively.

In the nine patients with CTV of 200–300 cm^3^, the range of FLV ratio in MV was 7.9%–48.5% (median value, 22.0%) and the range width (maximum FLV ratio in MV/minimum FLV ratio in MV) was 6.1. The maximum value (48.5%) of FLV ratio in MV was obtained in one patient with RILD.

In the three patients with CTV of 500–600 cm^3^, the range of FLV ratio in MV was 11.0%–17.6% (median value, 15.8%) and the range width was 1.6. The maximum value (17.6%) of FLV ratio in MV was obtained in one patient with RILD.

In the two patients with CTV of 1100–1200 cm^3^, the range of the FLV ratio in MV was 7.7%–16.6% (median value, 12.2%) and the range width was 2.2. The maximum value (16.6%) of the FLV ratio in MV was obtained in one patient with RILD.

The three cases of RILD are located at the upper margin of the scatter distribution diagram.

### 3.2. Risk Factors Related to RILD

Table 3 shows the results of an analysis of risk factors related to RILD, comparing patient backgrounds and RT characteristics between 54 cases without RILD and three cases with RILD. Significant difference between the two groups for univariate analysis was found only for RIDFLV/FLV (%) (p < 0.001). The mean RIDFLV/FLV (%) of the three cases of RILD was 24.2 ± 1.5%.

Figure 2E shows the relation of the boundary lines between RIDFLV and FLV (dotted lines) to the four iso-dose lines from simulation CT and SPECT after 3DCRT (Figures 2C and 2D). In all patients, the boundary lines passed through the midspace between the 20 and 30 Gy isodose curves, in each slice.

## 4. Discussion

In the present study, SPECT using Tc-99m-GSA was used to obtain a functional image of the liver. When portal vein flow is preserved in the whole liver, normal liver might be regarded as functional liver. However, in cases of HCC with PVTT, a significant negative correlation was observed between the FLV ratio in MV before RT and CTV (p < 0.001), as shown in Figures 2 and Figure 3. In other words, the destruction of liver function in the margin area surrounding HCC plus PVTT showed an increase with increasing size of HCC with PVTT. This result probably reflects the fact that the degree of destruction of functional liver is more advanced in large HCC with PVTT. To ensure the safety of 3DCRT for HCC with PVTT, it is crucial to obtain diagnostic information regarding the exact distribution of functional liver and to use this information to guide radiation planning.

In previous reports of 3DCRT for HCC, Liang *et al.* reported that 17 of 109 cases experienced severe RILD, and that 13 of 17 died of RILD [13]; and Cheng *et al.* reported that 12 of 68 cases experienced Grade 3 and 4 RILD, and that half died of RILD [14]. In these two studies, normal liver volume distribution rather than FLV distribution was considered in radiation planning. The existence of dysfunctional liver prior to RT in the margin area does not imply a worsening of liver function after RT. As previously reported, we undertook to reduce RIDFL by taking advantage of this dysfunctional liver before RT as a kind of natural spacer in radiation treatment planning [4]. In the present series, three of 57 patients experienced transient RILD and no patient died. The three cases of RILD were identified only by laboratory tests and experienced no clinical symptoms, and improved after supportive treatment. We consider that radiation planning that takes into account FLV distribution enables treatment without causing severe RILD.

Univariate analysis identified RIDFLV/FLV (%) as the only risk factor related to RILD (p < 0.001) (Table 3) and RIDFLV/FLV (%) as more sensitive than RIDFLV (cm^3^). The cut-off value of RIDFLV/FLV for mild RILD was 24.2 ± 1.5%. The boundary line between RIDFL and FL most commonly lay in the midspace between the 20 and 30 Gy isodose curves. Therefore, we propose that FLV20Gy appears to coincide with RIDFLV/FLV (%) as a predictive indicator of RILD. A limitation of the present study is that multivariate analysis was difficult to conduct because of the small number of cases of RILD [15]. For this reason, we investigated FLV distribution in all patients using the FLV ratio in M V. The three cases of RILD, with CTVs of 287, 587, and 1184 cm^3^, had the greatest FLV ratio in MV among the nine patients with CTV of 200–300 cm^3^, the three patients with CTV of 500–600 cm^3^, and the two patients with CTV of 1100–1200 cm^3^, respectively. In short, of the cases with the similar CTV, these three cases had the greatest functional liver which was destroyed by RT. It implies that the recognition of functional liver distribution is necessary for radiation planning to avoid severe RILD. A large discrepancy in range width (maximum FLV ratio in MV/minimum FLV ratio in MV) was observed among patients with similar CTVs. This discrepancy probably depends on the differing degrees of portal blood flow loss and/or dysfunctional liver caused by PVTT. The range width increased as the value of CTV decreased (Figure 3). This discrepancy exists not only in MV but in the whole liver. It reflects the latent risk of 3DCRT without recognizing functional liver. Namely, the main beam by radiation planning with referring to the SPECT image can be created to avoid functional liver as little as possible. Meanwhile, the radiation plan without utilizing the SPECT image potentially causes the greater damage to functional liver. Actually, before 2004 at our institute, two of the 59 cases who underwent 3DCRT without SPECT experienced severe RILD and died of RILD [16]. SPECT is the only method of obtaining information regarding FLV distribution. Referring to Figure 3, radiation planning can be modulated. Namely, if the planning data of CTV and FLV ratio in MV distributed beyond the upper limit in Figure 3, precaution against RILD would arise and main beam selection to lessen FLV would be conducted.

## 5. Conclusions

As the main tumor and PVTT (CTV) increased in size, we observed a decrease in the FLV ratio in MV before RT. With the two high-dose beams planned to irradiate FLV as little as possible, SPECT-B3DCRT yielded a destructive ratio to 24.2 ± 1.5%. Although 26/56 (45.6%) of the present series were Child–Pugh grade B, RILD was uncommon (3/57, 5.3%); in the three cases of RILD, the degree of RILD was mild and transient. Among patients with similar CTVs, those with RILD had the greatest FLV in MV. Radiation planning that into account takes the distribution of FLV appears to result in the least possible RILD.

## Figures and Tables

**Table 1.** Radiation-induced liver disease (RILD) [7].

Classic RILD	• Anicteric hepatomegaly and ascites

	• Elevation of alkaline phosphatase level to at least twice the pretreatment value or the upper limit of the normal range

Non-classic RILD	• Elevation of liver transaminases more than five times the upper limit of the normal range or CTCAE Grade 4 levels in patients with baseline transaminases values more than five times the upper limit of the normal range

	• Decline in liver function (defined as a worsening of Child–Pugh score by 2 or more)

Abbreviations: CTCAE = Common Terminology Criteria for Adverse Events v 3.0.

**Table 2.** Patient characteristics.

**Characteristic**	**Value**
**Age(y)**	

median	68
range	49–87
≤60	18 (31.6)
>60	39 (68.4)

**Gender**	

male	47 (82.5)
female	10 (17.5)

**Performance status**	

0	19 (33.3)
1	28 (49.1)
2	10 (17.5)

**Hepatitis virus type**	

HBV	14 (24.6)
HCV	33 (57.9)
both	4 (7.0)
unknown	6 (10.5)

**Child–Pugh grade**	

A	31 (54.4)
B	26 (45.6)

**AFP(IU/mL)**	

<400	32 (56.1)
≥400	25 (43.9)

**Apex of PVTT**	

1st branch	30 (52.6)
Main trunk	27 (47.4)

*Abbreviations*: PVTT = portal vein tumor thrombosis; HBV = hepatitis B virus; HCV = hepatitis C virus; AFP = α-fetoprotein. Data presented as number of patients with percentages in parentheses.

**Table 3.** Comparison of backgrounds and RT characteristics in the patients between with radiation-induced liver disease (RILD) and without.

**Characteristics**	**RILD**		**p**

	**absence (n = 54)**	**Presence (n = 3)**	
Gender			0.446 *
Male	45	2	
Female	9	1	
Age(y)			1.000 *
≦60	17	1	
>60	37	2	
Performance status			0.076 *
0–1	46	1	
2	8	2	
Apex of PVTT			0.599 *
1st branch	29	1	
Main trunk	25	2	
HBV			0.544 *
No	36	3	
Yes	18	0	
AFP (IU/mL)			1.000 *
<400	30	2	
≧400	24	1	
Child-Pugh grade			0.089 *
A	31	0	
B	23	3	
Clinical target volume (cm^3^)	301.4 ± 486.7	686.0 ± 456.5	0.187 †
Normal liver volume (cm^3^)	1096.1 ± 283.7	836.0 ± 163.9	0.123 †
Functional liver volume (cm^3^)	928.0 ± 275.8	697.5 ± 207.7	0.161 †
Radiation-induced dysfunctional liver volume (cm^3^)	122.3 ± 53.5	166.9 ± 40.2	0.162 †
Radiation-induced dysfunctional liver volume/functional liver volume (%)	13.2 ± 4.6	24.2 ± 1.5	<0.001 †

*Abbreviations*: RT = Radiotherapy; RILD = radiation-induced liver disease; PVTT = portal vein tumor thrombus; HBV = hepatitis B virus; AFP = α-feto protein. Data presented as number of patients or mean ± standard deviation.

* Fisher's exact test.

† Student's t test.

## References

[1] Yamada R., Sato M., Kawabata M., Nakatsuka H., Nakamura K., Takashima S. (1983). Hepatic artery embolization in 120 patients with unresectable hepatoma. Radiology.

[2] Lo C.M., Ngan H., Tso W.K., Liu C.L., Lam C.M., Poon R.T., Fan S.T., Wong J. (2002). Randomized controlled trial of transarterial lipiodol chemoembolization for unresectable hepatocellular carcinoma. Hepatology.

[3] Shirai S., Sato M., Suwa K., Kishi K., Shimono C., Kawai N., Tanihata H., Minamiguchi H., Nakai M. (2009). Single photon emission computed tomography-based three-dimensional conformal radiotherapy for hepatocellular carcinoma with portal vein tumor thrombus. Int. J. Radiat. Oncol. Biol. Phys..

[4] Shirai S., Sato M., Suwa K., Kishi K., Shimono C., Sonomura T., Kawai N., Tanihata H., Minamiguchi H., Nakai M. (2010). Feasibility and efficacy of single photon emission computed tomography-based three-dimensional conformal radiotherapy for hepatocellular carcinoma 8 cm or more with portal vein tumor thrombus in combination with transcatheter arterial chemoembolization. Int. J. Radiat. Oncol. Biol. Phys..

[5] Shuke N., Aburano T., Nakajima K., Yokoyama K., Sun B.F., Matsuda H., Muramori A., Michigishi T., Tonami N., Hisada K. (1992). The utility of quantitative 99 m Tc-GSA liver scintigraphy in the evaluation of hepatic functional reserve: Comparison with 99 mTc-PMT and 99mTc-Sn colloid. Kaku Igaku.

[6] Nanashima A., Yamaguchi H., Shibasaki S., Morino S., Ide N., Takeshita H., Tsuji T., Sawai T., Nakagoe T., Nagayasu T. (2006). Relationship between CT volumetry and functional liver volume using technetium-99m galactosyl serum albumin scintigraphy in patients undergoing preoperative portal vein embolization before major hepatectomy: A preliminary study. Dig. Dis. Sci..

[7] Pan C.C., Kavanagh B.D., Dawson L.A., Li X.A., Das S.K., Miften M., Ten Haken R.K. (2010). Radiation-associated liver injury. Int. J. Radiat. Oncol. Biol. Phys..

[8] Sawamura T., Nakada H., Hazama H., Shiozaki Y., Sameshima Y., Tashiro Y. (1984). Hyperasialoglycoproteinemia in patients with chronic liver disease and/or liver cell carcinoma. Gastroenterology.

[9] Park W., Lim D.H., Paik S.W., Koh K.C., Choi M.S., Park C.K., Yoo B.C., Lee J.E., Kang M.K., Park Y.J. (2005). Local radiotherapy for patients with unresectable hepatocellular carcinoma. Int. J. Radiat. Oncol. Biol. Phys..

[10] Emami B., Lyman J., Brown A., Coia L., Goitein M., Munzenrider J.E., Shank B., Solin L.J., Wesson M. (1991). Tolerance of normal tissue to therapeutic irradiation. Int. J. Radiat. Oncol. Biol. Phys..

[11] Park H.C., Seong J., Han K.H., Chon C.Y., Moon Y.M., Suh C.O. (2002). Dose-response relationship in local radiotherapy for hepatocellular carcinoma. Int. J. Radiat. Oncol. Biol. Phys..

[12] Mornex F., Girard N., Beziat C., Kubas A., Khodri M., Trepo C., Merle P. (2006). Feasibility and efficacy of high-dose three-dimensional-conformal radiotherapy in cirrhotic patients with small-size hepatocellular carcinoma non-eligible for curative therapies—Mature results of the French phase II RTF-1 trial. Int. J. Radiat. Oncol. Biol. Phys..

[13] Liang S.X., Zhu X.D., Xu Z.Y., Zhu J., Zhao J.D., Lu H.J., Yang Y.L., Chen L., Wang A.Y., Fu X.L. (2006). Radiation-induced liver disease in three-dimensional conformal radiation therapy for primary liver carcinoma: The risk factors and hepatic radiation tolerance. Int. J. Radiat. Oncol. Biol. Phys..

[14] Cheng JC-.H., Wu J.K., Huang C.M., Liu H.S., Huang D.Y., Cheng S.H., Tsai S.Y., Jian J.J., Lin Y.M., Cheng T.I. (2002). Radiation-induced liver disease after three-dimensional conformal radiotherapy for patients with hepatocellular carcinoma: Dosimetric analysis and implication. Int. J. Radiat. Oncol. Biol. Phys..

[15] Motulsky H. (1995). Intuitive Biostatistics.

[16] Shirai S., Kishi K., Shimono C., Kimura S., Tanihata H., Kawai N., Sato M., Sonomura T. (2006). Radiation therapy (RT) for hepatocellular carcinoma (HCC) with portal tumor thrombi (PVTT) of main portal vein and/or the 1^st^ branch. J. Clin. Radiol..

